# The Largest Outbreak of Acute Gastroenteritis of Mixed Norovirus Genogroups in the Coast of São Paulo State, Brazil

**DOI:** 10.3390/v18050555

**Published:** 2026-05-13

**Authors:** Rita de Cássia Carmona, Simone Guadagnucci, Mayara Esteves, Carla Costa, Simone Blotta, Daniele de Morais, Bráulio Machado, Cecilia Martins, Christiane Ristori, Ruth Rowlands, Damaris Pinto, Vitória de Souza, Bernadete Liphaus, Alessandra Xavier, Maria Inês Sato, Mikaela Barbosa, Ronalda de Araújo, Vanessa Cardoso, Luciano Candido, Renan Silva, Audrey Cilli

**Affiliations:** 1 Núcleo de Doenças Entéricas, Centro de Virologia, Instituto Adolfo Lutz, Secretaria de Estado da Saúde de, São Paulo 01246-900, Brazil; sgmorillo@gmail.com (S.G.); braulio.machado@ial.sp.gov.br (B.M.); audrey.chirelli@ial.sp.gov.br (A.C.); 2 Núcleo de Microbiologia, Centro de Alimentos, Instituto Adolfo Lutz, Secretaria de Estado da Saúde de, São Paulo 01246-900, Brazil; cecilia.martins@ial.sp.gov.br (C.M.); christiane.costa@ial.sp.gov.br (C.R.); ruth.rowlands@ial.sp.gov.br (R.R.);; 3 Divisão de Doenças de Transmissão Hídrica e Alimentar, Centro de Vigilância Epidemiológica “Prof. Alexandre Vranjac”, Secretaria de Estado da Saúde de São Paulo, São Paulo 01246-900, Brazilbliphaus@saude.sp.gov.br (B.L.); almfranco@saude.sp.gov.br (A.X.); 4 Departamento de Análise Ambiental, Companhia Ambiental do Estado de São Paulo (CETESB), São Paulo 05459-010, Brazil; misato@sp.gov.br (M.I.S.); mrbarbosa@sp.gov.br (M.B.);

**Keywords:** acute diarrhea, gastroenteritis, genotype, norovirus, outbreak, rotavirus, one health

## Abstract

During the Brazilian summer, from 29 December 2024, to 6 March 2025, a large cluster of acute gastroenteritis (AGE) outbreaks was reported along the coast of São Paulo State, Brazil, peaking in January 2025. Overall, 55 outbreaks involving 755 cases were officially notified, while more than 76,000 medical consultations for AGE were recorded across the region during the same period. A total of 50 stool samples were analyzed by RT-qPCR for group A rotavirus (RVA) and norovirus (NoV). NoV was detected in 27 samples (54.0%), confirming it as the main etiological agent, while RVA was identified in one sample (2.0%). Among NoV-positive cases, genogroup II (GII) predominated (59.0%), followed by genogroup I (GI) (19.0%) and mixed infections (22.0%). Genomic sequencing successfully genotyped 23 strains (95.8%), revealing six distinct genotypes. The recombinant GII.17[P17] was predominant (48.0%), followed by GI.3[P3], GI.3[P13], GI.5[P5], GII.4 Sydney_2012[P16], GII.3[P30], as well as mixed infections. No enteric viruses were detected in drinking water. However, seawater samples showed high concentrations of NoV GI and GII (up to 10^4^ GC L^−1^) at beaches unsuitable for bathing. Wastewater surveillance revealed high viral loads, particularly NoV GII (up to 10^8^ GC L^−1^), consistent with reported cases. To our knowledge, this is the first report in Brazil of a NoV-associated AGE outbreak investigated through an integrated approach combining clinical, environmental, and epidemiological surveillance data. Findings highlight genotype diversity and reinforcing the importance of integrated surveillance.

## 1. Introduction

Norovirus (NoV) is the leading cause of acute gastroenteritis (AGE) worldwide, and responsible for approximately 90% of sporadic cases and half of the outbreaks in all age groups [1,2,3]. Although AGE occurs at any age group, severe disease is common in children under five years old and in the elderly [4,5,6]. NoV accounts for an estimated 16–18% of global AGE cases, translating to nearly 685 million infections annually and approximately 210,000 deaths, including over 70,000 fatalities among children living in low-resource regions [1,7]. These data emphasize the susceptibility of developing settings and reinforce the urgent need for strengthening preventive strategies [7,8].

NoV, a member of the *Caliciviridae* family, is a non-enveloped, positive-sense, single-stranded RNA virus with a genome of approximately 7.5 kb [7,9]. The viral genome comprises three open reading frames (ORFs). ORF1 encodes the nonstructural proteins, including the RNA-dependent RNA polymerase (RdRp); ORF2 encodes the major capsid protein VP1; and ORF3 encodes the minor structural protein VP2 [7,9].

Noroviruses are classified using a binary system that combines the RdRp genotype (ORF1), designated by the letter “P”, with the VP1 capsid genotype (ORF2), both within a defined genogroup (G). This dual nomenclature reflects the genetic composition of these genomic regions and is particularly useful for identifying recombinant strains, thereby providing a more accurate characterization of NoV genetic diversity [7].

Phylogenetic analysis of complete VP1 amino acid sequences has classified NoV into 10 genogroups (GI–GX). Among these groups, GI, GII, GIV, GVIII, and GIX infect humans (referred to as HuNoV) [7,9,10]. To date, more than 48 genotypes have been identified across these genogroups and 60 polymerase genotypes (P-types) based on partial RdRp nucleotide sequences [7,9]. HuNoV is dominated by GI and GII, with at least 9 GI genotypes (GI.1–GI.9) and 27 GII genotypes (GII.1–GII.27) recognized. GII.4 genotype, in particular, has been responsible for the majority of global outbreaks over the past three decades, frequently evolved into new pandemic variants [7,9,11,12]. The remaining genogroups—GIV (e.g., GIV.1, GIV.2), GVIII, and GIX—are less prevalent in humans but contribute to sporadic cases. Animal-associated genogroups include GIII in cattle and sheep, GV in mice, GVII in dogs, and GX in cats, highlighting the wide range of NoV hosts and the potential for cross-species transmission [7,9,13].

NoV outbreaks are most often associated with enclosed environments such as hospitals, long-term care facilities, schools, and childcare centers, where person-to-person is the main transmission route [14,15]. However, community outbreaks have also been reported, particularly following ingestion of contaminated food or water, or at recreational seawater activities [16,17].

The National Program for Monitoring Acute Diarrhea was implemented in Brazil by the Ministry of Health in 1994. This syndromic surveillance system records community cases of acute diarrhea and includes sample collection and case analysis to monitor the epidemiological trends, promptly detect variations indicative of outbreaks, and thus guiding prevention and control measures. It is important to emphasize that outbreaks of AGE are subject to mandatory reporting in Brazil [18,19].

In this context, NoV outbreaks have increasingly emerged as a significant public health concern in Brazil, with a marked rise in reported cases over recent years [20,21,22,23,24,25]. Therefore, this study describes the largest outbreak of AGE reported on the coast of São Paulo State, Brazil, associated with the co-circulation of multiple NoV genogroups and genotypes.

## 2. Materials and Methods

### 2.1. Outbreaks Description

From 29 December 2024 to 6 March 2025, corresponding to the summer and rainy season in the Southern Hemisphere, outbreaks of AGE were reported in the South and North Coast of São Paulo State, Brazil, through both the National Program for Monitoring Acute Diarrhea and the Notifiable Diseases Information System (SINAN).

A suspected case was defined as an individual presenting with ≥3 episodes of acute watery diarrhea within 24 h, with or without associated symptoms such as vomiting, fever, or abdominal pain, residing in or visiting the affected municipalities during the study period.

### 2.2. Clinical Samples

A total of 50 stool samples were collected from patients with AGE who sought medical care during outbreak investigations. The samples originated from multiple outbreaks occurring in geographically distinct municipalities along the coast of São Paulo State, Brazil. Samples were sent to the Enteric Diseases Laboratory, Virology Center, Adolfo Lutz Institute, São Paulo State, a Regional Reference Laboratory for Rotavirus and Viral Enteric Infection Surveillance under the Brazilian Ministry of Health, for virological investigations.

### 2.3. Sample Processing and RNA Extraction

Stool suspensions (10%, *w*/*v*) were prepared in Tris-calcium buffer, pH 7.2 and clarified by centrifugation. Viral genomes were extracted from 200 µL of stool suspensions (10%, *w*/*v*) using the Extracta 16 automated system (Extracta 16, Loccus, Cotia, Brazil) with the EXTRACTA KIT GOLD—DNA and RNA Viral (Loccus^®^, Cotia, São Paulo, Brazil). All procedures were carried out according to the manufacturer’s instructions.

### 2.4. Screening for RVA and NoV by RT-qPCR

RVA screening was performed using an in-house TaqMan-based one-step RT-qPCR assay targeting the NSP3 gene, as described by Zeng et al. [26]. NoV genogroups GI and GII were detected with an in-house TaqMan-based one-step duplex RT-qPCR assay targeting the ORF1/2 junction region, according to Hill et al. and Kageyama et al. [27,28]. In each assay, negative controls and human stool samples previously confirmed positive for RVA and NoV were used.

### 2.5. NoV Genomic Sequencing

NoV-positive samples identified by RT-qPCR were further analysed by conventional one-step RT-PCR for dual genotyping of the polymerase and capsid regions. Reactions were carried out using the SuperScript™ III One-Step RT-PCR Kit (Invitrogen, Carlsbad, CA, USA) with primer sets Mon 432/G1SKR for GI and Mon 431/G2SKR for GII, targeting the ORF1/2 junction region [29,30]. The resulting amplicons (543 bp for GI and 557 bp for GII) were purified with the PureLink™ PCR Purification Kit (Invitrogen) and subjected to sequencing using the BigDye™ Terminator v3.1 Cycle Sequencing Kit (Applied Biosystems, Foster City, CA, USA) with the same primers used for amplification. Sequencing was performed on ABI 3500 Genetic Analyzer (Applied Biosystems), and chromatograms were edited manually in Sequencher™ v4.1.4 (Gene Codes Corporation, Ann Arbor, MI, USA). Genotypes were assigned using the Norovirus Typing Tool v1.12 (https://www.genomedetective.com/app/typingtool/nov/ (accessed on 5 October 2025)).

### 2.6. Phylogenetic Analysis

Phylogenetic analysis was performed using the Maximum Likelihood (ML) method under the Kimura two-parameter model [31]. The best-supported tree was selected based on the highest log-likelihood value. Node support values are indicated as the percentage of replicate trees in which the corresponding clusters were recovered.

Initial tree topologies were generated using Neighbor-Joining (NJ) and BioNJ algorithms from a pairwise distance matrix estimated by the Maximum Composite Likelihood (MCL) method, with the topology showing the highest likelihood retained. All analyses were conducted in MEGA X software v. 10.2 [32].

### 2.7. Nucleotide Sequence Accession Numbers

All representative NoV sequences obtained in this study were deposited in the GenBank database under accession numbers PX104391 to PX104397 (GI), PX104350 to PX104364 (GII), PX093044 (GI-coinfection) and PX093053 (GII-coinfection).

### 2.8. Viral Differential Diagnosis

In parallel, all samples were subjected to differential diagnostic testing for the detection of other enteric viruses, including human adenoviruses (HAdV), enteroviruses (EVs), and human sapoviruses (HSaV). Detection was carried out using in-house TaqMan-based one-step RT-qPCR singleplex assays [33,34,35].

### 2.9. Environmental Investigation

#### 2.9.1. Drinking-Water Samples

Four drinking-water samples were collected from the public supply systems of the municipalities of Praia Grande and Guarujá, located on the southern coast of the State of São Paulo, Brazil. Sampling was performed by the local Sanitary Surveillance authority using 1.5 L polyethylene bottles at each sampling point. The samples were transported under refrigeration in insulated coolers to the Microbiology Laboratory, Food Center, Adolfo Lutz Institute, São Paulo, Brazil.

Viral concentration was carried out according to Katayama et al. (2002) [36] with modifications. A 1.5 L aliquot of each sample was filtered through a 0.45 µm pore-size membrane connected to a vacuum pump system. Prior to filtration, magnesium chloride hexahydrate (MgCl_2_•6H_2_O) was added and the sample pH was adjusted to 5.0. Subsequently, 300 mL of 0.5 mM H_2_SO_4_ (pH 3.0) was filtered. The membrane was transferred to a Petri dish and eluted with 15 mL of 1 mM NaOH (pH 10.5) under agitation for 10 min; this procedure was performed twice. The eluate obtained was transferred to a Falcon tube to which 50 µL of 50 mM H_2_SO_4_ and 50 µL of 100× TE buffer was added. The entire recovered volume was centrifuged at 1500× *g* for 15 min at 4 °C; this centrifugation step was repeated twice. The final concentrated volume obtained was 2 mL.

Genomic RNA was extracted using the QIAamp^®^ Viral RNA Mini Kit (Qiagen, Hilden, Germany) and the Extracta 16 automated system (Loccus, Cotia, Brazil) with the EXTRACTA KIT GOLD—DNA and RNA Viral (Loccus^®^, Cotia, Brazil). All procedures were performed according to the manufacturers’ instructions.

NoV genogroups GI and GII screening was performed using an in-house TaqMan-based one-step duplex RT-qPCR assay targeting the ORF1/2 junction region, according to Kageyama et al. (2003) [27].

#### 2.9.2. Seawater Samples—Bathing Beaches

Seawater samples (1.5 L each) were collected and processed by the laboratories of the Department of Environmental Analyses at CETESB (Environmental Company of the State of São Paulo, Brazil). Samples were collected in polypropylene bottles, transported on ice, stored at 4–8 °C, and processed within 24 h of collection [37,38].

Four seawater samples were collected from beaches located in the municipalities of Santos, Praia Grande e Guarujá. The beaches were selected because they frequently receive unsuitable bathing classifications in the weekly bathing water quality-monitoring program conducted by CETESB [39,40], which is based on the enumeration of *Enterococcus* spp. In addition, these beaches receive a high number of beachgoers, particularly during the summer season. The Tombo Beach in Guarujá, which has been awarded the Blue Flag certification, was used as a reference control site due to its recognized excellent water quality. According to Brazilian environmental legislation [41], beaches are classified as unsuitable for bathing when *Enterococcus* concentrations exceed 100 CFU/100 mL.

For viral analysis, 250 mL of seawater were centrifuged at 3500× *g* for 30 min to remove suspended solids. The supernatant was subsequently concentrated by ultrafiltration using two Centricon^®^ Plus-70 centrifugal filters (10 kDa molecular weight cutoff) (Merck Millipore, Burlington, MA, USA) per sample. The filters were centrifuged at 3000× *g* for 20 min until the concentrate volume was reduced to approximately 300 µL. Viral con-centrates were recovered by centrifugation at 1000× *g* for 2 min, according to the manufacturer’s instructions.

Marine water samples were also analysed for *Enterococcus* spp. as indicators of fecal contamination, using the membrane filtration (MF) method [42,43].

Additionally, viral concentrates from seawater samples were subjected to a PMAxx™ viability assay (Biotium Inc., Freemont, CA, USA), as described by Stobnicka-Kupiec et al. (2022) [44], allowing the selective detection of intact (potentially viable) viruses. Briefly, 6 µL of PMAxx™ reagent (previously diluted to 2 mM) were added to 200 µL of the viral concentrates. After homogenization, samples were incubated in the dark, under agitation (300 rpm) at 24 °C for 20 min. Subsequently, samples were transferred to a photoactivation device (PMA-Lite™ LED Photolysis Device, Biotium) and exposed to LED light (40 W, 460 nm) for 20 min to activate the dye. After brief centrifugation, samples were subjected to nucleic acid extraction.

#### 2.9.3. Wastewater Samples

CETESB conducts wastewater-based environmental surveillance at several locations across the state of São Paulo. In the Baixada Santista region, two monitoring points that primarily receive wastewater from Santos and São Vicente (Santos Outfall) and São Sebastião (Santiago Pumping Station) were evaluated for RVA, NoV, EVs, and HAdV during the outbreaks period (December 2024 to March 2025).

Raw sewage samples (1.5 L), collected as described in 2.9.2, were concentrated by membrane adsorption–filtration, based on the method described by Ahmed et al. (2015) [45]. Briefly, 1 mL of MgCl_2_ (2.5 M) was added to 100 mL of wastewater sample. The pH was adjusted to 3.0–3.5 using 1 M HCl to enhance viral adsorption to the membrane. The sample was filtered through an electronegative membrane filter (0.45 µm pore size, HAWP Millipore, 90 mm) placed in a Büchner funnel connected to a vacuum filtration system. After filtration, membranes were removed and cut into small fragments using sterile scissors and forceps. The membrane pieces were transferred to 5 mL microcentrifuge tubes for subsequent nucleic acid extraction.

For viral DNA/RNA extraction, 200 μL of the concentrated samples were processed using the AllPrep PowerViral DNA/RNA Kit (Qiagen, Hilden, Germany) according to the manufacturer’s instructions. The final elution was performed in 100 μL of RNase-free ultrapure water.

Viral genome quantification was performed by TaqMan one-step RT-qPCR, using the TaqPath™ 1-Step RT-qPCR Master Mix (Applied Biosystems, Foster City, CA, USA) for RNA virus and TaqMan^®^ Environmental Master Mix 2.0 (Applied Biosystems, Foster City, CA, USA) for HAdV.

RVA was detected using an RT-qPCR assay targeting the NSP3 gene [26]. NoV GII and GII were detected using a duplex RT-qPCR assay targeting the ORF1-ORF2 junction region [46]. HAdV and EV were quantified by qPCR assays targeting the hexon gene and the 5′ untranslated region (5′ UTR), respectively, according to previously described methods [47,48].

### 2.10. Ethical Statement

This study was conducted in accordance with the principles of the Declaration of Helsinki and was approved by the Ethics Committee of the Adolfo Lutz Institute (approval date: 20 October 2025; Certificate of Ethical Review Submission [CAAE] number 91636825.1.0000.0059).

## 3. Results

### 3.1. Outbreaks of AGE

In total, 55 outbreaks were notified, involving 755 cases. In addition, from 29 December 2024 to 6 March 2025, 76,504 medical consultations for AGE were recorded in the region. Therefore, surveillance data revealed a sustained community transmission.

**Figure 1:**
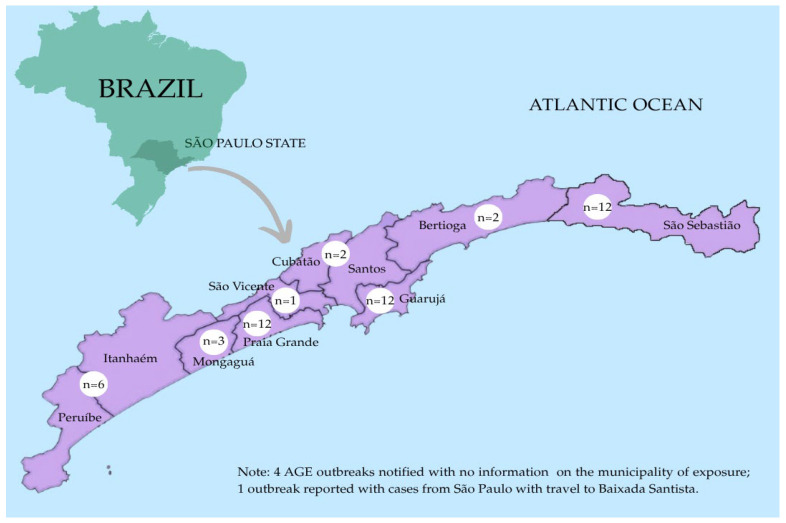
Distribution of AGE outbreaks reported between December 2024 and March 2025 along the coastline of São Paulo State, including the Baixada Santista region, the southern coast, and the northern coast municipalities, Bertioga and São Sebastião. Legend: “n” refers to the number of reported outbreaks in each municipality.

During the study period, 10 municipalities were affected: Bertioga, Cubatão, Guarujá, Itanhaém, Mongaguá, Peruíbe, Praia Grande, Santos, São Vicente, and São Sebastião (Figure 1).

In addition to diarrhea, the patients involved in the outbreaks had fever, nausea, vomiting, abdominal pain, and headache. Most cases were mild, requiring only oral hydration. Five cases were hospitalized, and two resulted in death.

### 3.2. RVA and NoV Detection

A total of 50 stool samples were analysed from patients presenting with AGE during the outbreaks period. NoV was the predominant viral agent detected, being identified in 27 (54.0%) of the analysed samples. Among the NoV-positive cases, genogroup GII was the most frequent (16/27; 59.3%), followed by GI/GII mixed infections (6/27; 22.2%) and GI alone (5/27; 18.5%). In contrast, RVA was detected in only 1 sample (2.0%); however, genotyping was not performed due to the extremely low viral load (cycle threshold, Ct > 30), which prevented successful amplification. For NoV-positive patients, the age distribution ranged from 10 months to 68 years, with a mean age of 36.4 years and a median age of 31 years. The sex distribution showed a predominance of males (16/27; 59.3%) compared to females (11/27; 40.7%).

The mean age of patients with clinical samples collected was 31.4 years (median age of 30 years; range: from 5 months to 70 years), with a balanced sex distribution (25 females and 25 males).

### 3.3. NoV Molecular Characterization by Genomic Sequencing

Among the 27 samples NoV-positive, 24 with Ct values ≤ 30 were selected for genomic sequencing, as lower Ct values indicate higher viral loads suitable for amplification. Of these, 23 (95.8%) yielded sufficient sequence data for successful genotyping. Within GII, the GII.17[P17] strain predominated, being identified in 12 cases (52.2%, n = 12/23), followed by single detections of GII.4 Sydney_2012[P16] (1 case/23; 4.3%) and GII.3[P30] (1 case/23; 4.3%). Among GI strains, three samples (13.0%) were classified as GI.3[P3] and two GI.3[P13] (8.7%). Mixed infections displayed diverse combinations, including GI.3[P3]/GII-No typing (1 case; 4.3%), GI.5[P5]/GII-No typing (1 case; 4.3%), GI-No typing/GII.17[P17] (1 case; 4.3%), and mix GI.3[P3]/GII.17[P17] (2 cases; 8.7%).

### 3.4. Phylogenetic Analysis of NoV GI and GII Strains

Partial sequences of the RdRp and VP1 capsid regions were successfully obtained from the 23 NoV-positive. Phylogenetic reconstruction combined with nucleotide identity analysis revealed the co-circulation of genetically distinct lineages belonging to both GI and GII, including recombinant strains.

#### 3.4.1. NoV GII—RdRp Genotypes and Capsid

##### RdRp Phylogeny and Polymerase Genotypes

**Figure 2:**
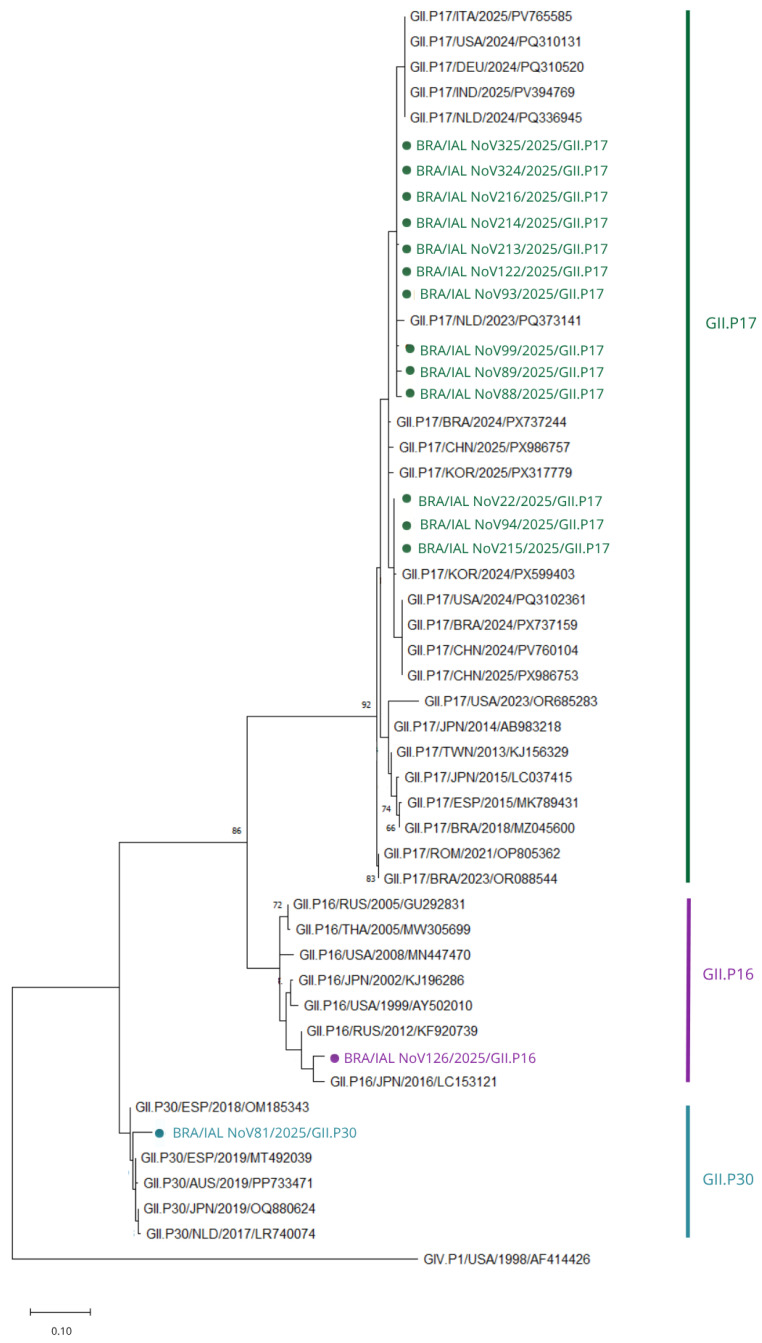
Phylogenetic tree based on partial RdRp sequences of NoV GII. Brazilian strains clustered predominantly within GII.P17, with additional clustering in GII.P16 and GII.P30. The tree was constructed using the neighbor-joining method under the Kimura 2-parameter model with gamma distribution (K2 + G) and 2000 bootstrap replicates. Only bootstrap values > 65 are shown. The scale bar indicates substitutions per site. Sequences obtained in this study are highlighted in green (GII.P17), purple (GII.P16) and blue (GII.P30).

Phylogenetic analysis of the RdRp region showed that the majority of GII sequences clustered within the GII.P17 polymerase genotype, forming a well-supported clade with contemporary global strains (Figure 2). Representative Brazilian sequences grouped closely with recent reference strains, confirming their classification within a currently circulating lineage.

Pairwise nucleotide identity analysis demonstrated very high similarity among Brazilian GII.P17 sequences (>99%), indicating limited intra-lineage variability. The highest nucleotide identities (up to 99.6%) were observed among Brazilian strains (BRA/2018/MZ045600, BRA/2023/OR088544, BRA/2024/PX737159, BRA/2024/PX737244) and a contemporary strain from South Korea (KOR/2024/PX599403). Other international reference strains, including those from North America and Europe, showed slightly lower identities (approximately 98–99%), consistent with the widespread global circulation of this lineage.

In addition to the predominant P17 lineage, two additional polymerase genotypes were identified: GII.P30 and GII.P16. A single strain clustered within the P30 lineage, clearly separated from the P17 and P16 clades, while another strain grouped within the P16 lineage, forming a distinct cluster consistent with globally distributed P16 strains.

For the GII.P30 strain, nucleotide identities ranged from approximately 96–97% with contemporary international sequences, with lower values (~92–93%) observed for older reference strains. The closest matches included strains from Europe and Oceania, particularly the Netherlands (NLD/2017/LR740074), Spain (ESP/2019/MT492039; ESP/2018/OM185343), and Australia (AUS/2019/PP733471).

For the GII.P16 strain, nucleotide identity values ranged from approximately 95–97% compared with contemporary strains, with the highest similarities observed with sequences from Russia, Japan, and the United States, supporting its classification within a globally circulating P16 lineage.

##### VP1 Phylogeny and Capsid Genotypes

**Figure 3:**
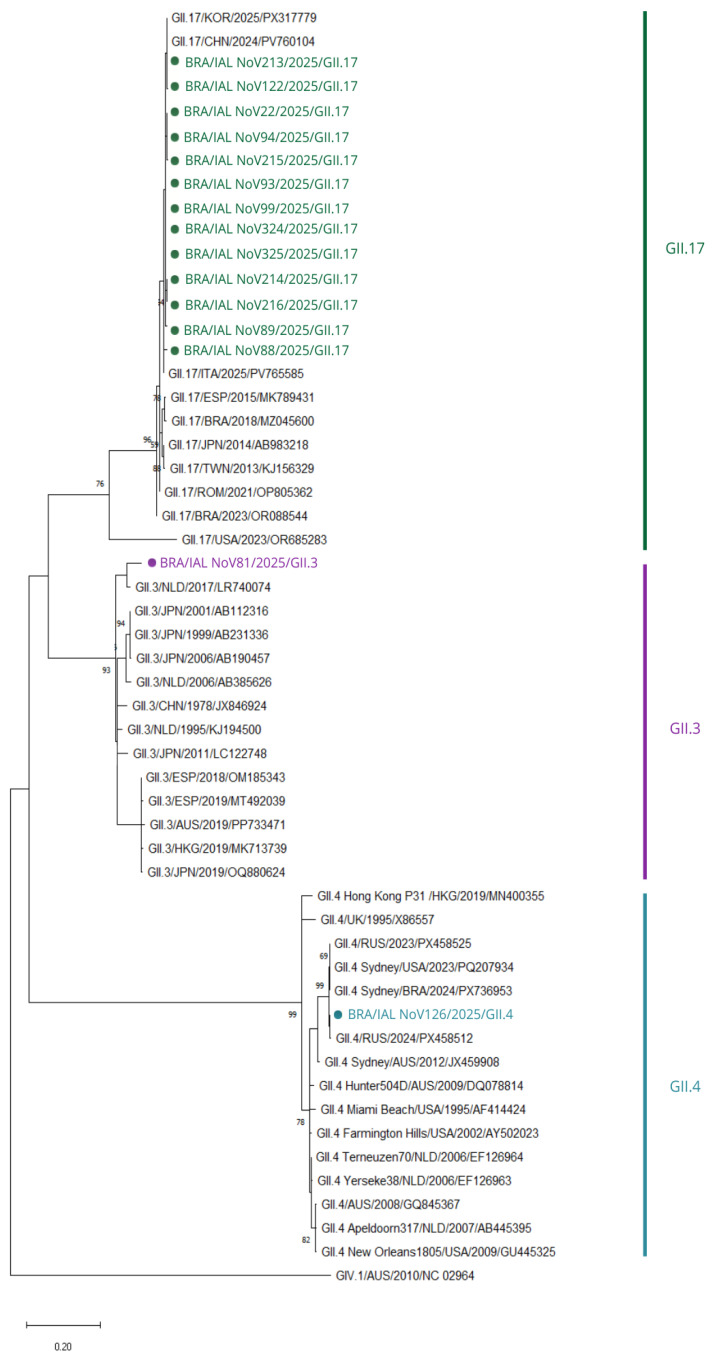
Phylogenetic tree based on partial VP1 sequences of NoV GII. Brazilian strains clustered predominantly within GII.17, with additional clustering in GII.3, and GII.4 Sydney. The tree was constructed using the neighbor-joining method under the Kimura 2-parameter model with gamma distribution (K2 + G) and 2000 bootstrap replicates. Only bootstrap values > 65 are shown. The scale bar indicates substitutions per site. Sequences obtained in this study are highlighted in green (GII.17), purple (GII.3) and blue (GII.4).

Analysis of the VP1 region identified three capsid genotypes: GII.17, GII.3, and GII.4 Sydney, with sequences clustering according to their respective lineages as shown in Figure 3.

The predominant RdRp lineage (GII.P17) was associated with the GII.17 capsid genotype, forming a well-supported cluster with contemporary strains from multiple geographic regions. Nucleotide identities ranged from approximately 96% to 98.6%, with the highest similarity observed with strains from Italy (ITA/2025/PV765585) and China (CHN/2024/PV760104), whereas lower values were associated with older reference sequences.

The GII.P30 polymerase strain was associated with the GII.3 capsid genotype. VP1 nucleotide identity values ranged from approximately 94% to 96%, with the closest matches to strains from Japan (JPN/2019/OQ880624), Australia (AUS/2019/PP733471), and Spain (ESP/2019/MT492039), indicating relatedness to recently circulating GII.3 variants (circa 2019).

The GII.P16 polymerase strain was associated with the GII.4 Sydney capsid genotype. In the VP1 phylogeny, this sequence clustered with contemporary Sydney variants, showing the highest nucleotide identities (~98–99%) with recent strains from Russia (RUS/2023/PX458525; RUS/2024/PX458512), the United States (USA/2023/PQ207934), and Brazil (BRA/2024/PX736953), while lower similarities (~94–96%) were observed when compared with older variants.

##### Combined RdRp–VP1 Classification

Combined analysis demonstrated the co-circulation of three recombinant GII constellations: GII.17[P17], GII.3[P30], and GII.4 Sydney[P16]. The predominant lineage, GII.17[P17], showed strong genetic relatedness to strains circulating in Asia, Europe, and North America, indicating extensive global connectivity.

#### 3.4.2. NoV GI—RdRp Genotypes and Capsid

##### RdRp Phylogeny and Polymerase Genotypes

**Figure 4:**
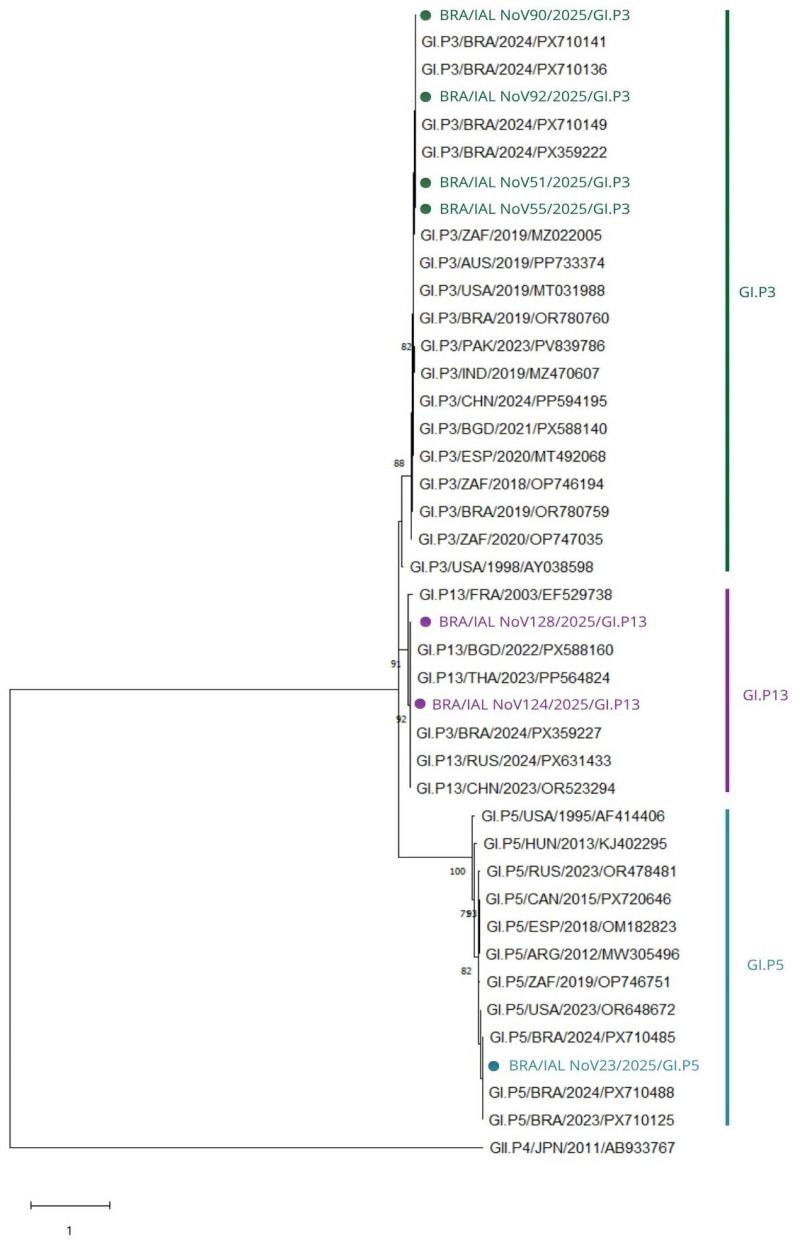
Phylogenetic tree based on partial RdRp sequences of NoV GI. Brazilian strains are distributed among GI.P3, GI.P5, and GI.P13, including one divergent GI.P13 sequence. The tree was constructed using the neighbor-joining method under the Kimura 2-parameter model with gamma distribution and invariant sites (K2 + G + I) with 2000 bootstrap replicates. Only bootstrap values > 65 are shown. The scale bar indicates substitutions per site. Sequences obtained in this study are highlighted in green (GI.P3), purple (GI.P13) and blue (GI.P5).

Phylogenetic analysis of the GI RdRp region resolved the Brazilian sequences into three distinct polymerase genotypes: GI.P5, GI.P3, and GI.P13 (Figure 4).

The strain BRA/IAL NoV23/2025 clustered within the GI.P5 lineage, whereas BRA/IAL NoV51/2025, BRA/IAL NoV55/2025, BRA/IAL NoV90/2025, and BRA/IAL NoV92/2025 formed a compact and well-supported cluster within the GI.P3 lineage. Two additional strains, BRA/IAL NoV124/2025 and BRA/IAL NoV128/2025, grouped within the GI.P13 lineage.

For the GI.P5 strain, nucleotide identity analysis revealed the highest similarity (98.5–99.6%) with recent Brazilian sequences (BRA/2024/PX710488; BRA/2023/PX710125). Among international strains, the closest matches were from the United States (96.7%), followed by South Africa (95.2%), Argentina (93.8%), Canada (93.4%), and Spain (93.0%), supporting its classification within a contemporary GI.P5 lineage.

The GI.P3 cluster showed high intra-lineage similarity (97.8–99.2%), indicating low genetic divergence. The closest international matches included strains from South Africa (97.4–97.8%), the United States (97.0–97.4%), and China (up to 97.0%), with additional related strains from Pakistan, Bangladesh, India, Spain, and Australia showing identities between approximately 93.0% and 96.7%. These findings are consistent with a conserved and widely circulating GI.P3 lineage.

The GI.P13 strains showed two distinct patterns. The sequence BRA/IAL NoV124/2025 exhibited high nucleotide identity with contemporary reference strains, including those from Russia and Thailand (98.9%), Bangladesh (98.5%), and China (97.0%), confirming its classification within a recent GI.P13 lineage. In contrast, BRA/IAL NoV128/2025 displayed markedly lower identities across all available references, with the highest similarity to a Chinese strain (73.0%), followed by Russia and Thailand (70.5%), suggesting a highly divergent partial RdRp sequence.

##### VP1 Phylogeny and Capsid Genotypes

**Figure 5:**
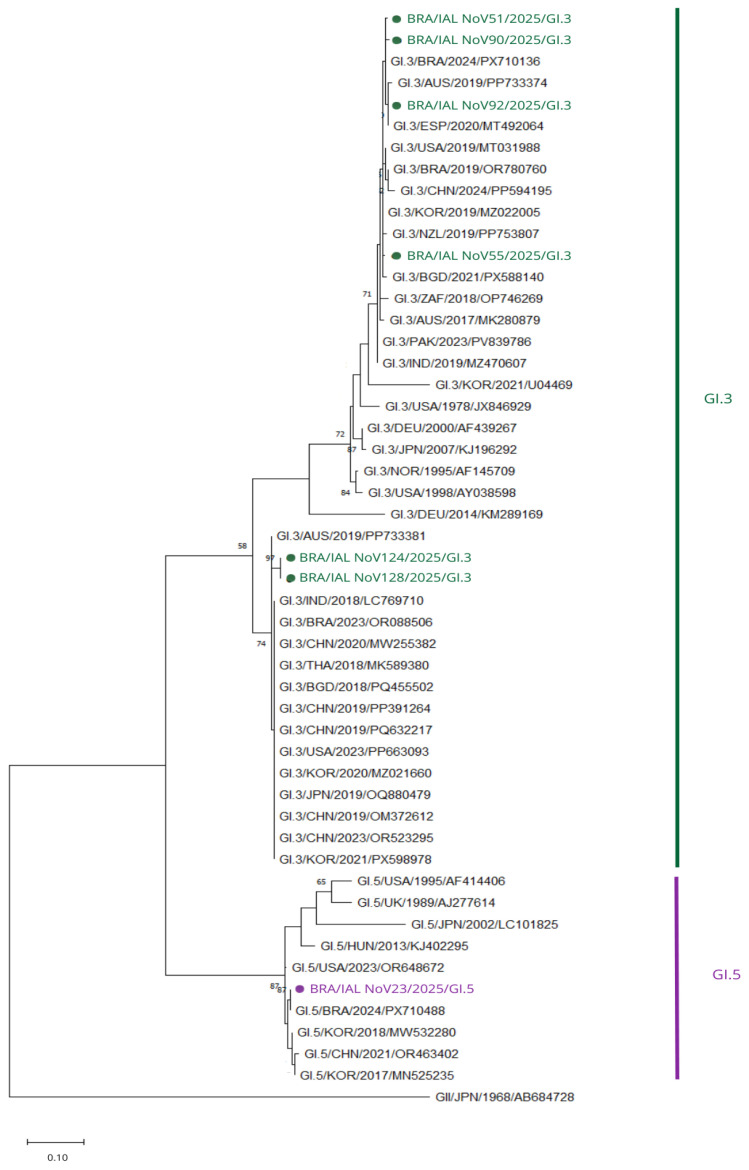
Phylogenetic tree based on partial VP1 sequences of NoV GI. Brazilian strains cluster within GI.3 and GI.5 and are closely related to contemporary global strains. The tree was constructed using the neighbor-joining method under the Kimura 2-parameter model with gamma distribution and invariant sites (K2 + G + I) with 2000 bootstrap replicates. Only bootstrap values > 65 are shown. The scale bar indicates substitutions per site. Sequences obtained in this study are highlighted in green (GI.3), purple (GI.5).

Analysis of the VP1 region identified two capsid genotypes among the GI strains: GI.5 and GI.3 (Figure 5).

The strain BRA/IAL NoV23/2025 clustered within the GI.5 capsid lineage, showing the highest nucleotide identity with a recent Brazilian strain (100%; BRA/2024/PX710488), followed by strains from South Korea (~99.0%; KOR/2017/MN525235; KOR/2018/MW532280), the United States (98.9%; USA/2023/OR648672), and China (98.5%; CHN/2021/OR463402). Lower similarities were observed with older GI.5 strains from Europe and Japan, indicating its placement within a contemporary lineage.

The remaining strains clustered within the GI.3 capsid genotype. Four sequences (BRA/IAL NoV51/2025, BRA/IAL NoV55/2025, BRA/IAL NoV90/2025, and BRA/IAL NoV92/2025) formed a highly homogeneous cluster, with the highest identities observed with strains from Brazil, South Korea, Spain, and the United States (up to 99.2–100%). Additional similarities were observed with strains from Pakistan, India, and Australia.

Two sequences (BRA/IAL NoV124/2025 and BRA/IAL NoV128/2025) were identical to each other in the VP1 region (100%) and showed the highest similarity (~98.2%) with strains from China, Japan, South Korea, the United States, Thailand, and Bangladesh, confirming their classification within the GI.3 lineage.

##### Combined RdRp–VP1 Classification

Combined analysis revealed three GI genetic constellations: GI.5[P5], GI.3[P3], and GI.3[P13].

The strain BRA/IAL NoV23/2025 showed concordant classification as GI.5[P5], while four strains (BRA/IAL NoV51/2025, BRA/IAL NoV55/2025, BRA/IAL NoV90/2025, and BRA/IAL NoV92/2025) were consistently classified as GI.3[P3].

In contrast, BRA/IAL NoV124/2025 and BRA/IAL NoV128/2025 were classified as GI.3[P13], reflecting a discordant RdRp–VP1 combination consistent with a recombinant genomic constellation. Phylogenetic analysis further demonstrated that these recombinant GI.3[P13] strains clustered separately within the VP1 tree, forming a distinct sub-cluster within the broader GI.3 lineage.

### 3.5. Detection of Other Enteric Viruses

Differential diagnostic testing was conducted to assess the presence of other enteric viruses using singleplex real-time PCR assays targeting HAdV, EVs, and HSaV. HAdV DNA was detected as a single pathogen in 6 out of 50 samples (12.0%), whereas EV RNA was identified as a sole agent in 2 out of 50 samples (4.0%). Viral co-detections were observed in 11/50 cases (22.0%). Among the co-infected samples, NoV plus HAdV was identified in 4/11 cases (36.4%; 8.0% overall), NoV plus EVs in 3/11 cases (27.3%; 6.0% overall), HAdV plus EVs plus HSaV in 3/11 cases (27.3%; 6.0% overall), and EVs plus HSaV in 1/11 case (9.1%; 2.0% overall) (Table S1).

### 3.6. Environmental Investigation

#### 3.6.1. Drinking-Water Samples

NoV was not detected in any of the analysed water samples, and neither HAdV nor RVA. Additionally, no enteric viruses were detected in public water supply samples from Praia Grande and Guarujá. These findings indicate the absence of detectable viral contamination at the time of sampling and may reflect the effectiveness of water treatment and disinfection processes.

#### 3.6.2. Seawater Samples—Bathing Beaches

Seawater samples from beaches in Guarujá and Praia Grande were collected on two occasions in early 2025, coinciding with the period with the highest number of reported cases of AGE in the Baixada Santista region. RVA, NoV GI, NoV GII, EV, and HAdV were detected in the sample collected at Enseada Beach (Guarujá) on 5 January 2025. NoV GI and GII were quantified at concentrations of 5.07 × 10^4^ and 5.59 × 10^4^ GC L^−1^, respectively. During the sampling week, Enseada Beach (Guarujá) was classified as unsuitable for bathing according to the CETESB bathing water-monitoring program, which is consistent with the elevated *Enterococcus* concentration observed in the seawater sample. During the February 2025 sampling campaign, viable HAdV was detected across all beaches, except for Tombo Beach, which was considered the control site. The RT-qPCR results for enteric viruses, together with the bacterial indicator *Enterococcus*, are presented in Table S2.

#### 3.6.3. Wastewater (Sewage) Samples

Enteric viruses were consistently detected in wastewater samples from both monitoring sites throughout the study period, with NoV GII and HAdV showing the highest concentrations. In São Sebastião, NoV GII reached concentrations close to 10^8^ GC L^−1^, while NoV GI remained relatively stable between 10^6^ and 10^7^ GC L^−1^. RVA presented lower concentrations, generally ranging between 10^3^ and 10^5^ GC L^−1^. HAdV showed consistently high concentrations, whereas EVs exhibited a gradual increase over the monitoring period. Similar patterns were observed in Santos/São Vicente, where NoV GII predominated and EVs concentrations increased towards the end of the study period (Figure S1).

## 4. Discussion

This study describes the largest recorded cluster of AGE outbreaks in the coastal region of São Paulo State, Brazil, characterized by extensive community transmission and the co-circulation of multiple NoV genogroups and genotypes. The integration of epidemiological, molecular, and environmental data provides a comprehensive understanding of the outbreaks dynamics and highlights the multifactorial nature of transmission in coastal settings. Importantly, this event had a substantial public health and socioeconomic impact, widely reported in the media, including a marked increase in healthcare demand, over burdening of emergency services, and significant effects on tourism, with cancellations and reduced visitor flow in affected municipalities. In addition, national news outlets confirmed the role of NoV as the etiological agent of the outbreaks based on laboratory findings from the Adolfo Lutz Institute, further amplifying public awareness and concern (https://agenciabrasil.ebc.com.br/saude/noticia/2025-01/surto-de-virose-no-litoral-paulista-foi-provocado-por-norovirus, accessed on 5 October 2025).

In Brazil, despite the growing data of literature on NoV epidemiology, large-scale community outbreaks integrating clinical, molecular, and environmental data remain poorly documented. Most available studies are limited to single-source outbreaks or hospital-based surveillance, with scarce evidence linking viral genetic diversity to environmental contamination pathways [20,21,22,23,24,25]. In this context, the present study provides novel insights by combining epidemiological surveillance, high-resolution molecular characterization, and environmental investigation to elucidate the dynamics of a large-scale outbreaks.

NoV was identified as the primary etiological agent, being detected in more than half of the analysed samples, whereas RVA was identified only sporadically. This finding is consistent with the well-established role of NoV as the leading cause of AGE outbreaks worldwide across all age groups [2,5,7,8,15,16]. The predominance of adult cases observed in this study, particularly among individuals aged 20–50 years, aligns with previous reports indicating that NoV affects all age groups, although more severe outcomes are typically observed in young children and elderly populations [14,15,16].

Molecular characterization revealed a high genetic diversity of circulating strains, with the predominance of the recombinant genotype GII.17[P17], followed by the detection of GII.4 Sydney[P16], GII.3[P30], and multiple GI genotypes, including GI.3[P3], GI.5[P5], and GI.3[P13]. The predominance of genogroup II strains corroborates previous studies demonstrating that GII viruses are responsible for the majority of outbreaks globally [1,9].

The predominance of GII.17[P17] in these outbreaks is epidemiologically significant. The co-detection of multiple recombinant genotypes and mixed infections further indicates a complex transmission network, a pattern seldom reported in South America [24,49,50,51]. These findings are consistent with recent evidence from Brazil, where GII.17[P17] was implicated as the predominant lineage in a large outbreak in Santa Catarina, with environmental transmission linked to extreme weather events [24]. Since its initial detection in Brazil in 2015 [52], GII.17[P17] has demonstrated sustained circulation and epidemic potential. Historically, this genotype has been associated with rapid expansion in Asia, where it temporarily displaced GII.4 as the dominant lineage in countries such as China, Japan, and Taiwan [53,54,55].

Although GII.4 variants have long been the principal drivers of global NoV pandemics [12,15,16], accumulating evidence indicates a marked epidemiological shift. Recent surveillance data from the United States reveal a dramatic increase in GII.17-associated outbreaks, rising from <10% in the 2022–2023 season to approximately 75% in 2024–2025, overtaking GII.4 and coinciding with an earlier seasonal onset [56]. Parallel trends have been reported across Europe and North America, where GII.17 accounted for 17–64% of GII detections during the 2023–2024 season, alongside a decline in GII.4 prevalence [57]. Phylogenetic analyses indicate that most circulating strains cluster with lineages first described in Romania in 2021, with the emergence of novel sub-lineages, underscoring ongoing viral diversification and cross-border dissemination [57].

Similar dynamics have been observed in Asia, particularly in China during the 2024–2025 season, where GII.17 accounted for up to 62% of NoV-positive cases, supplanting GII.4 [58]. Interestingly, these strains share a recent common ancestor with those circulating in Europe and the United States, pointing to rapid global spread of closely related variants [58].

Recent evidence suggests that the successful re-emergence of GII.17[P17] is associated with dynamic and adaptive evolutionary processes, particularly involving amino acid substitutions and structural changes in the VP1 capsid protein that may enhance binding affinity to histo-blood group antigens (HBGAs) and promote antigenic diversification, favoring immune evasion and sustained transmission in human populations [59]. Tohma et al. [59] demonstrated that contemporary GII.17 variants underwent extensive diversification during the early epidemic phase, followed by the stabilization of adaptive lineages associated with efficient human transmission. These findings provide a plausible evolutionary explanation for the persistence and competitive fitness of GII.17 alongside globally predominant GII.4 variants.

The high nucleotide similarity of GII.17[P17] observed among Brazilian strains and their clustering with contemporary global sequences suggest recent introduction and rapid dissemination. These findings reinforce the role of global viral circulation and population mobility in shaping NoV epidemiology [2,5].

In the Latin American context, NoV outbreaks in the recent past have been predominantly associated with GII.4 variants, particularly in countries such as Brazil, Argentina, and Chile, with only limited reports of large outbreaks driven by emerging non-GII.4 genotypes [22,23,24,60,61]. Therefore, the predominance of GII.17[P17] observed in this study suggests ongoing shifts in the molecular epidemiology of NoV in South America.

The detection of recombinant strains, including GII.3[P30] and GI.3[P13], further supports the importance of recombination as a major evolutionary mechanism in NoV diversification [5,9,12]. Recombinant strains have been increasingly associated with outbreaks and may exhibit altered transmissibility or fitness. Recent evidence from Brazil further supports this mechanism. A foodborne outbreak in Espírito Santo State in 2023 was associated with a rare recombinant genotype, GII.10[P16], with identical sequences detected among affected individuals, indicating a common source of infection. The high similarity of these strains to previously reported international sequences suggests recent introduction and highlights the ability of uncommon recombinant genotypes to cause outbreaks [25]. Additionally, the identification of mixed GI/GII infections indicates high exposure levels and provides opportunities for recombination events, further contributing to viral diversity.

The genetic diversity observed in this study also has important implications for NoVVaccine development. Current vaccine candidates are primarily based on virus-like particles derived from predominant genotypes, particularly GII.4; however, the increasing circulation of non-GII.4 genotypes, such as GII.17 and recombinant strains, may affect cross-protective immunity and vaccine effectiveness [3,5,7]. This shift has important implications for NoV epidemiology, as well as for vaccine design and effectiveness, given the antigenic diversity of emerging strains. Sustained genomic surveillance and integrated epidemiological analyses will be critical to anticipate future epidemic trajectories and inform public health responses [62].

The co-circulation of multiple genotypes and the occurrence of mixed infections, as observed in these outbreaks, further highlight the challenges in achieving broad and durable protection. These findings reinforce the need for continuous molecular surveillance to inform vaccine design and to ensure that emerging variants circulating in regions such as Brazil and other Latin American countries are adequately represented in future immunization strategies [5,7].

The detection of other enteric viruses, including HAdV, EVs, and HSaV, particularly in co-infections, indicates the concurrent circulation of multiple pathogens during the outbreaks period. However, their lower detection frequency compared to NoV supports a secondary role in outbreak dynamics, consistent with previous reports [16,34,35].

From an epidemiological perspective, surveillance data demonstrated sustained community transmission, with 55 outbreaks and more than 76,000 medical consultations recorded during the study period. This magnitude reflects the high transmissibility of NoV and the influence of environmental and seasonal factors [2,15,16]. The outbreaks occurred during the summer and rainy season, a period characterized by increased tourism, higher population density in coastal areas, and elevated precipitation levels, all of which contribute to environmental contamination, facilitating viral transmission.

Environmental investigation can support a multifactorial transmission scenario. Initially, a common-source hypothesis related to drinking water was considered. However, NoV was not detected in treated water samples. Studies conducted in Brazil have reported a low frequency of NoV detection in treated waters, although temporary failures in treatment systems or extreme weather events may result in sporadic contamination episodes [63,64]. The sensitivity of the adsorption–elution method employed and the relatively small sample volume processed (1.5 L) may have limited detection of low viral concentrations [36,65,66]. Therefore, negative results should be interpreted with caution.

Although NoV was not detected in treated water, environmental investigation was essential to rule out the public water supply as a potential source of the outbreaks. This finding shift attention to other possible transmission routes, such as contaminated food, recreational waters, or person-to-person contact.

In contrast, seawater samples analysed by CETESB, which conducts routine monitoring of bathing water quality, showed the presence of all target viruses, including NoV GI and GII, at Enseada Beach (January 2025). Noroviruses exhibited the highest concentrations, highlighting their persistence in environmental waters. Viral occurrence was consistent with *Enterococcus* spp. levels [41,67], reinforcing the importance of routine monitoring for assessing bathing water quality.

Coastal waters are strongly influenced by the discharge of treated and untreated urban wastewater, as well as by combined sewer overflows during rainfall events. Rainfall levels along the coast of São Paulo State were significantly higher than the historical average during December 2024 and January 2025 [40]. Consequently, exposure to contaminated recreational waters likely represents an additional transmission pathway and may have contributed to the increased incidence of AGE disease in the evaluated coastal region.

Wastewater surveillance of enteric virus circulation in Santos, São Vicente, and São Sebastião had already indicated the presence of NoV GI and GII in early December 2024, followed by an increase in January 2025, coinciding with the rise in reported clinical cases. A progressive increase in EVs concentrations was also observed, which might have contributed to the intensification of the viral AGE outbreaks. Overall, these findings highlight the importance of environmental surveillance of enteric viruses as a complementary tool for monitoring fecal contamination and supporting public health risk assessment.

The number of clinical samples analysed depicts only a small proportion of the total reported cases, which may limit the representativeness of the detected NoV genotypes and the overall genetic diversity circulating during the outbreaks.

Although RT-qPCR combined with Sanger sequencing proved effective for the rapid identification and molecular characterization of circulating NoV strains during this outbreak, these approaches have inherent limitations in detecting the full genetic complexity of mixed infections and recombinant viruses. Partial sequencing of the ORF1/ORF2 junction region provides important genotyping information; however, it may underestimate intra-host viral diversity and limit the identification of minor variants and recombination breakpoints. In this context, future investigations using High-Throughput Sequencing (NGS) and whole-genome sequencing (WGS) approaches will be essential to achieve higher-resolution molecular epidemiological analyses. These strategies may allow more accurate reconstruction of transmission chains, improved detection of co-infections and recombination events, and a better understanding of the evolutionary dynamics driving the emergence and spread of novel NoV variants during large community outbreaks.

From a public health perspective, these findings have important implications for outbreaks preparedness and response strategies. The evidence of multifactorial transmission, involving environmental contamination and widespread community exposure, underscores the need for integrated surveillance systems that combine epidemiological, laboratory, and environmental data. Strengthening wastewater-based surveillance and routine monitoring of recreational waters may provide early warning signals for viral circulation and support timely interventions.

In addition, the observed genetic diversity and circulation of recombinant NoV strains reinforce the importance of continuous molecular surveillance within national reference networks. Improvements in sanitation infrastructure, particularly in coastal regions subject to seasonal population increases, and optimization of water treatment processes, are critical for reducing transmission risk. Collectively, these measures are essential to enhance resilience against future outbreaks and to mitigate the public health burden of NoV-associated AGE.

## 5. Conclusions

This study highlights the multifactorial nature of NoV-associated AGE outbreaks in coastal settings, driven by the co-circulation of diverse genotypes and environmental contamination. The predominance of emerging and recombinant strains, including GII.17[P17], underscores the dynamic evolution of NoV and its implications for surveillance and control.

These findings reinforce the importance of integrating epidemiological, clinical, molecular, and environmental data to support effective public health responses. Strengthening molecular surveillance, improving sanitation, and expanding environmental monitoring are essential to reduce transmission risk. Although no licensed vaccine is currently available, ongoing vaccine development efforts highlight the need for continuous molecular surveillance to inform future immunization strategies and improve outbreak preparedness in Brazil and similar settings.

## Data Availability

The consensus sequence of the viruses analysed in this study was submitted to the GenBank database under the accession numbers PX093044, PX093053, PX104350 to PX104364, PX104391 to PX104397.

## Supplementary Materials

The following supporting information can be downloaded at: https://www.mdpi.com/article/10.3390/v18050555/s1, Figure S1: Temporal trends of enteric viruses detected in wastewater samples collected in São Sebastião and Santos/São Vicente between December 2024 and March 2025. Open symbols represent values below the limit of detection (LOD); Non-detected values were replaced by LOQ/2, while values below the limit of quantification (LOQ) were substituted by LOQ/√2; Table S1: Distribution of viral co-infections detected in stool samples during norovirus-associated diarrhea outbreaks in the coastal region of São Paulo State, Brazil; Table S2: Detection of enteric viruses and Enterococcus in seawater samples collected from beaches in Baixada Santista, São Paulo, Brazil.
