# Supplementary material for: The Largest Outbreak of Acute Gastroenteritis of Mixed Norovirus Genogroups in the Coast of São Paulo State, Brazil
Source: Viruses. 2026 May 13;18(5):555. doi: 10.3390/v18050555 (PMC13211592; doi:10.3390/v18050555)
Supplement: Supplementary file 1 [file viruses-18-00555-s001.zip › Supplementary Figure S1.pdf]

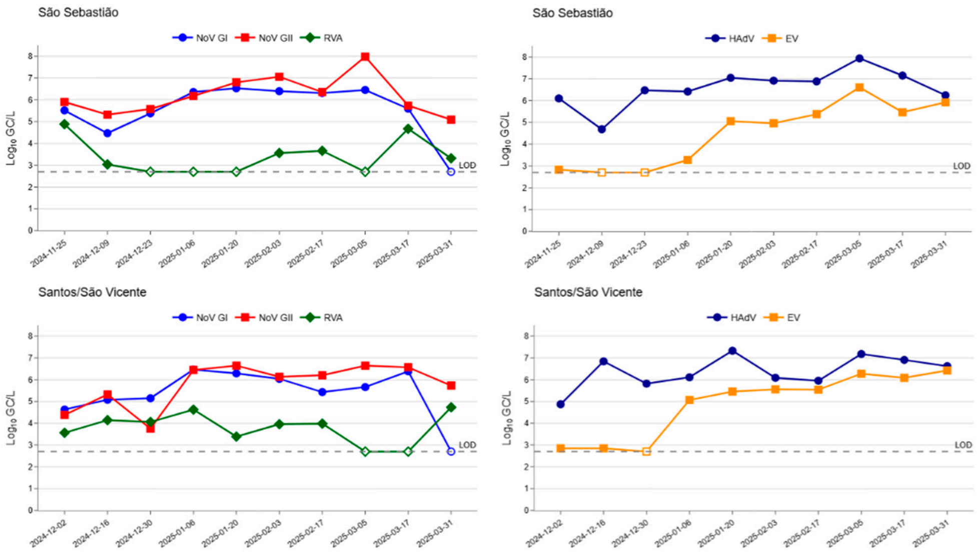

Figure S1. Temporal trends of enteric viruses detected in wastewater samples collected in São Sebastião and Santos/São Vicente between December 2024 and March 2025. Open symbols represent values below the limit of detection (LOD); Non-detected values were replaced by LOQ/2, while values below the limit of quantification (LOQ) were substituted by LOQ/√2.
