# Supplementary material for: The Largest Outbreak of Acute Gastroenteritis of Mixed Norovirus Genogroups in the Coast of São Paulo State, Brazil
Source: Viruses. 2026 May 13;18(5):555. doi: 10.3390/v18050555 (PMC13211592; doi:10.3390/v18050555)
Supplement: Supplementary file 1 [file viruses-18-00555-s001.zip › Supplementary Tables S1 and S2.pdf]

Table S1: Distribution of viral co-infections detected in stool samples during norovirus-associated diarrhea outbreak in the coastal region of São Paulo State, Brazil, 2025.

Patient ID	Age of patient	Sex	Co-Infection
22	60 years old	F	NoV GI/GII + EVs
88	30 years old	F	NoV GII + HAdV
96	39 years old	M	NoV GI/GII + HAdV
99	49 years old	M	NoV GII + HAdV
126	10 month old	M	NoV GII + EVs
216	51 years old	M	NoV GII + HAdV
274	61 years old	F	NoV GII + EVs
353	1 year old	F	EVs + HSaV
354	11 months old	F	HAdV + EVs + HSaV
355	2 years old	F	HAdV + EVs + HSaV
356	1 year old	F	HAdV + EVs + HSaV

ID: Identification, F: Female, M: Male

Table S2: Detection of enteric viruses and *Enterococcus* in seawater samples collected from beaches in Baixada Santista, São Paulo, Brazil.

Sampling date	Sampling site	PMAxx-(RT)-qPCR (GC L^{-1})					MF (CFU mL^{-1})
		RVA	NoV GI	NoV GII	EV	HAdV	*Enterococcus*
05 Jan 2025	Enseada Beach, Guarujá	Detected	5.07×10^4	5.59×10^4	3.19×10^3	1.71×10^3	640
23 Feb 2025	Enseada Beach, Guarujá	ND	ND	ND	ND	1.77×10^3	6
23 Feb 2025	Gonzaga Beach, Santos	ND	ND	ND	ND	4.46×10^2	14
23 Feb 2025	Vila Mirim Beach, Praia Grande	ND	ND	ND	ND	4.98×10^2	4
23 Feb 2025	Tombo Beach, Guarujá	ND	ND	ND	ND	ND	5

ND = not detected (below the limit of detection); LOD (viruses) = 1.0×10^3 GC L^{-1}.
